# Orexin receptor type 2 agonism inhibits thermogenesis in brown adipose tissue by attenuating afferent innervation

**DOI:** 10.7555/JBR.36.20220033

**Published:** 2022-05-28

**Authors:** Mo-qiu Jia, Yong-jin Wang, Kang Fu, Han Jiao, Jia Sun, Yuanqing Gao

**Affiliations:** Key Laboratory of Cardiovascular and Cerebrovascular Medicine, School of Pharmacy, Nanjing Medical University, Nanjing, Jiangsu 211166, China

**Keywords:** orexin receptor type 2, brown adipose tissue, thermogenesis

## Abstract

Orexin signaling has been associated with energy expenditure and brown adipose tissue (BAT) function. However, conflicting data exist in the field about how orexin signaling regulates BAT thermogenesis. In this study, we show that a specific orexin receptor type 2 (OX2R) agonist [Ala11, D-Leu15]-OxB (OB-Ala) inhibited intrascapular brown adipose tissue (iBAT) thermogenesis by reducing sympathetic output to iBAT. This effect is mediated by OX2Rs located on afferent nerve endings innervating iBAT instead of brown adipocyte itself. Microinjection of OB-Ala into iBAT inhibited iBAT thermogenesis in mice upon cold exposure and neuronal activity in the paraventricular nucleus. Findings suggest that OB-Ala could inhibit iBAT thermogenesis by attenuating sensory input thereby inhibiting the sympathetic-sensory iBAT feedback loop. Our study uncovers a novel primary action site of orexin in the regulation of energy balance.

## Introduction

The high prevalence of obesity and associated morbidities is a worldwide public health issue in modern society. Understanding the mechanisms involved in obesity is crucial for metabolic research. Obesity is a consequence of disrupted energy balance, which could be influenced by many genetic and environmental factors. Orexin is a neuropeptide derived from the lateral hypothalamus and participates in energy balance regulation *via* two G protein-coupled receptors: Orexin receptor type 1 (OX1R) and orexin receptor type 2 (OX2R). Orexin-deficient mice become obese, and suffer hypophagia, and narcolepsy^[1]^, while the phenotypes of OX1R-deficient mice and OX2R-deficient mice are more complicated^[2]^. Therefore, detailed mechanisms underlying the orexin signaling network requires further investigation.

Brown adipose tissue (BAT) is responsible for thermogenesis and contributes to energy expenditure. In experimental mice, the induction of BAT activity has proven to be beneficial for obesity^[3]^. Therefore, BAT has attracted the attention of obesity researchers for decades. Orexin signaling also plays a role in body temperature regulation and thermogenesis^[4]^. Infusion of orexin in the brain induces sympatheoexcitatory effects, increasing heart rate and the heat production of BAT^[5]^. Orexin-deficient mice are accompanied by thermogenesis failure^[6]^. The loss of function suggests that the enhanced energy expenditure phenotype of orexin overexpression mice is more likely to be mediated by OX2R rather than OX1R^[7]^. However, there are also some contradictory reports regarding orexin signaling and BAT functions. For example, Sellayah *et al* reported that orexin could directly induce BAT differentiation during development *via* OX1R^[6]^, while another study claimed no abnormalities in BAT of orexin-deficient mice and a very low level of orexin receptors in BAT^[2]^. Some *in vitro* studies have suggested that OX1R and OX2R have the opposite effect on differentiation of preadipocytes into mature adipocytes^[8]^. Therefore, it is not known whether OX2R signaling directly regulates BAT function and we have yet to describe the underlying mechanisms.

BAT is innervated by both efferent and afferent nerves. Recent studies point out that sensory neurons also participate in BAT thermogenesis regulation. Sensory denervation achieved by systemic capsaicin infusion decreases thermogenic capacity and inhibits mitochondrial content^[9]^. Calcitonin gene-related peptide α in sensory neurons has been reported to participate in adaptive BAT thermogenesis regulation^[10]^. Meanwhile, orexin is also thought to mediate analgesic effects in dorsal root ganglion (DRG)^[11]^. Therefore, we hypothesize that orexin receptors in DRG which innervate BAT might be involved in BAT thermogenesis regulation by modulating primary afferent input from BAT.

In this study, we employed an OX2R specific agonist [Ala11, D-Leu15]-OxB (OB-Ala) to assess the influences of OX2R signing on systemic metabolism and BAT functions. We found that chronic treatment of OX2R agonist inhibits energy expenditure and suppresses BAT thermogenesis, which is accompanied by less sympathetic output. We also found out that OX2R is mainly expressed in afferent nerves, which innervates BAT instead of brown adipocytes. The sympathetic nerve denervation experiment further supports the notion that OX2Rs are located in afferent neurons. Microinjection of OB-Ala into BAT inhibits thermogenesis upon cold exposure. c-Fos activity was also lowered in the sympathetic neural modulation center paraventricular nucleus (PVN) in the OB-Ala group. Together, our data proved that activating OX2R on BAT afferent nerves inhibits sensory feedback to the brain and attenuates hypothalamic sympathetic outflow to BAT.

## Materials and methods

### Experimental animals

C57BL/6J mice, aged 8 to 12 weeks, were fed with a standard chow diet (Xietong Shengwu, China) and housed under a 12-hour light/dark cycle. All experimental procedures conformed to guidelines and protocols approved by Animal Core Facility of Nanjing Medical University (Approval No. IACUC-2006028 and IACUC-2202003).

For chronic i.p. injection, OB-Ala (16 nmol/kg, Tocris, USA) or an equal volume of saline was injected once daily for three weeks (*n*=7 for saline; *n*=9 for OB-Ala). For acute intra-bat injection, mice were anesthetized with 2% isoflurane, and 3 µL OB-Ala (0.3 nmol) were injected into intrascapular brown adipose tissue (*n*=6 for saline; *n*=7 for OB-Ala). Mice were then transferred to a 4 °C chamber five minutes after injection to ensure they had were fully recovered from anaesthesia. Intrascapular brown adipose tissue (iBAT) temperatures were recorded at a 1-minute interval by a pre-implanted thermal probe IPTT-300 (BioMedic Data System, Plexx, the Netherlands) between iBAT.

### Intracerebroventricular injection

Mice were anesthetized by i.p. injection of zoletil (50 mg/kg, Virbac, China) mixed with xylazine hydrochloride (10 mg/kg, Aladdin, China) and afixed by a stereotaxic instrument (RWD, China). The depth of anaesthesia was confirmed by a lack of flexor response to a toe pinch. Cannulas were placed in lateral ventricle with the coordination: +/− 1.2 mm (lateral), −0.6 mm (posterior), −2.2 mm (ventral) relative to Bregma. Mice were allowed to recover from surgery for at least one week before the experiment. When the body weight of mice was recovered to the baseline, mice received a 2 µL single injection of vehicle or OB-Ala (0.3 nmol) and were euthanized 90 minutes later for c-Fos analysis.

### Primary culture

iBATs were obtained from 0 to 2 day neonatal C57Bl/6J mice for primary brown adipocyte culture. Tissue was washed, minced and digested for 45 minutes at 37 °C in the isolation buffer containing 1× Hank's Balanced Salt Solution (Gibco, USA), 1.3 mmol/L CaCl_2_, 5 mmol/L glucose, 100 mmol/L N-2-Hydroxyethylpiperazine-N-2-ethanesulfonic acid (MDBio, China), 4% Bovine Serum Albumin (BSA; Beyotime, China) and 1.5 mg/mL collagenase I (Yeasen, China). The cell suspension was filtered through a 100 µm cell strainer, centrifuged at 600 *g* for 10 minutes, and resuspended in growth medium DMEM/F12 (Gibco) plus 1% Glutamax (Gibco), 1% penicillin and streptomycin (Gibco), and 10% fetal bovine serum (FBS; Gibco). Homogenate was filtered through a 40 µm cell strainer. Primary brown adipocytes were seeded in 12-well plates at 37 °C in a humidified 5% CO_2_ incubator.

Confluent cells were exposed to an adipogenic cocktail differentiation medium containing dexamethasone (5 µmol/L, MCE, USA), isobutylmethylxanthine (0.5 mmol/L, MCE), rosiglitazone (1 µmol/L, MCE), indomethacin (125 µmol/L, MCE), lithyronine (T3; 1 nmol/L, MCE), and insulin (5 µg/mL, Beyotime). Two days after induction, cells were maintained in an adipocyte culture medium containing T3 (1 nmol/L), insulin (5 µg/ mL) and rosiglitazone (1 µmol/L). On day 4 of differentiation, cells were switched to a growth medium containing T3 (1 nmol/L) and insulin (5 µg/mL) and ready for experiments. After serum starvation for 4 hours, cells were treated with saline or 10 nmol/L OB-Ala for 4 hours and then challenged by additional 0.5 µmol/L isoproterenol (ISO; Sigma, USA) or ddH_2_O for 4 hours before harvest. For lipid droplet staining, cells were fixed with 4% paraformaldehyde (PFA; Sigma) for 30 minutes at room temperature and then incubated in boron dipyrromethene (BODIPY; ThermoFisher Scientific, USA) for 20 minutes. 4′,6-diamidino-2-phenylindole was used to stain the cell nuclear.

Primary neurons of DRG were cultured according to methods described before^[12]^. In brief, DRGs (thoracic 1–10) were bilaterally dissected out from newborn C57BL/6J mice (5–8 days old) and digested for 45 minutes at 37 °C in an isolation medium containing 1 mg/mL collagenase typeⅡ (Yeasen) and 2.5 mg/mL dispase type Ⅱ (Yeasen) in DMEM/F12. Cells were centrifuged and resuspended twice with fresh medium after digestion and then seeded in a 6-well plate in a 37 °C incubator for 30 to 40 minutes to remove glial cells. The supernatant was centrifuged, resuspended, and seeded with a fresh medium consisting of 10% FBS, 1% Glutamax, 100 ng/mL NGF (Gibco) and DMEMF/12.

### iBAT denervation

Denervation experiments were conducted with 8 to 12 weeks old male C57BL/6J mice (*n*=4). Intact iBAT denervation was achieved through surgery^[13]^. Shortly, after anesthetization with isoflurane, the skin was cut from the middle of the BAT with a sterilized scalpel and the right side of the iBAT pat was entirely exposed by detaching the underlying tissues. All the nerves innervating the right BAT lobe were severed. The left side only was exposed without cutting the innervating nerves (sham surgery).

Mice were allowed to recover for one week before the experiment. Sympathetic denervation of iBAT was achieved using chemical methods *i.e.*, 6-hydroxydopamine (6-OHDA; Aladdin) based on the protocol described previously^[14–15]^. After anesthetization, 10 mg/mL 6-OHDA (in 1% vitamin C solution) was injected into both sides of iBAT pats, with four local injections per BAT pat and 2 µL per injection. An equal volume of 1% vitamin C was injected into the control group. Mice were allowed to recover for two weeks before the experiment to ensure sufficient denervation.

### iBAT and core body temperature

To measure iBAT temperature, thermal probes (IPTT-300) were implanted in the interscapular region between two fat pads under anaesthesia at least one week prior to commencing the experiment. A non-contact DAS-7007 reader was used for measurement. Rectal temperature was measured by a digital animal thermometer (Haorunqi Electronic Sci-tech Co., China). The digital thermometer was calibrated with thermal probes to ensure data consistency.

### Metabolic measurement

For metabolic phenotyping, mice were housed separately with an integrated intelligent behavior analysis system (IntelliCage by NewBehavior, TSE, Germany) on a 12-hour light/dark cycle (light: 8 a.m.–8 p.m.; dark: 8 p.m.–8 a.m.). Food intake, heat production, physical activity, and other metabolic parameters were monitored for 72 hours at 22 °C. Blood pressure was recorded with a non-invasive blood pressure analysis system (BP-2000, Visitech, USA) from the mouse tail. Ten to fifteen consecutive measurements were automatically performed after mice were adapted to the system.

### RNAscope*in situ* hybridization

*In situ* hypocretin receptor type 2 (*Hcrtr2*) was performed using a RNAscope Kit (Advanced Cell Diagnostics, China). Tissues were fixed in 4% PFA in 4 °C for 48 hours. After dehydration with 30% sucrose, samples were cryosectioned to 8- to 15-µm slices and then digested by protease plus at 40 °C for 30 minutes. *Hcrtr2* probe (NM 198962.3; Advanced Cell Diagnostics, USA) was used following the manufacturer's instruction. Images were acquired by LSM800 laser confocal microscope (Zeiss, Germany).

### Histological analysis

The inguinal white adipose tissue (iWAT) and iBAT were fixed in 4% PFA at 4 °C for 48 hours and then embedded using paraffin. The embedded tissues were sectioned into 8 µm, mounted on glass slides, and then stained with hematoxylin and eosin (H&E). Adipocyte size quantification method was described in image analysis section.

### Immunohistochemistry and immunofluorescence

Immunohistochemistry and immunofluorescence were carried out as previously described^[16]^. In brief, animals were euthanized with isoflurane inhalation and perfused with saline. Then, the entire brain was removed and fixed by 4% PFA for 48 hours, dehydrated in 30% sucrose, and then embedded and cut into 30 µm cryosections with optimal cutting temperature compound (Sakura, USA).

iBATs were also fixed in 4% PFA at 4 °C for 48 hours and then embedded with paraffin and cut into 8-µm paraffin section. Brain and iBAT sections were incubated by primary antibodies overnight at 4 °C. For diaminobenzidine staining, sections were subsequently incubated with biotin-conjugated secondary antibody followed by avidin-biotin-horseradish peroxidase complex and visualized by 1% diaminobenzidine with 0.01% hydrogen peroxide and then counterstained with hematoxylin. The stereotaxic coordinates of arcuate nucleus (ARC) and dorsal medial hypothalamus (DMH) brain sections: bregma −1.58 mm to −1.94 mm. The stereotaxic coordinates of PVN brain sections: bregma −0.82 mm to −0.70 mm.

For immunofluorescent staining, sections were incubated with Alexa dye conjugated secondary antibody and mounted with anti-fading mounting medium (Beyotime). The following primary and secondary antibodies with indicated dilutions were used in this study: anti-cFos (1:1000; Cell Signaling Technology, USA); anti-Tyrosine hydroxylase (1:500; Millipore, USA); anti-pro-opiomelanocortin (1:500; Abcam, UK); anti-agouti-related peptide (1:1000; R&D, USA), 488-AffiniPure donkey anti-rabbit IgG (1:400; Jackson, USA), 594-AffiniPure donkey anti-goat IgG (1:400; Jackson), biotin-conjugated donkey anti-rabbit IgG (1:400; Proteintech, USA).

Pericardial adipose tissue was freshly prepared as a whole mount sample on the glass slides and then fixed with 4% PFA for 1 hour at room temperature. The slides were incubated with BODIPY for 20 minutes at room temperature. Air-dried sections were mounted with an anti-fading mounting medium. Images were taken with a fluorescence microscope (Olympus, Japan) or LSM800 laser confocal microscope.

### Image analysis

Fiber intensity of pro-opiomelanocortin (POMC), agouti-related peptide (AGRP), tyrosine hydroxylase (TH), and c-Fos positive cell number were analyzed by Image J software (NIH, USA). The area covered by the immunostaining signals above the fixed threshold in the corresponding anatomical regions represents the "reactivity". Each coronal brain section was quantified by the average of the left side and the right side. Each brain sample was quantified by the average of two sections at the same anatomical level. The c-Fos positive cell was confirmed by visual recognition.

For adipocytes, the averaged cross-sectional area was estimated by the ratio between the volume density of adipocytes and twice the numerical density of adipocytes in a fixed area. Volume density of adipocytes was determined by point counting on a test system according to the literature^[17–18]^. Four fields per section were analyzed. Each adipocyte sample was quantified by the average of four sections.

### Western blotting

Fresh tissues were homogenized with appropriate volumes of RIPA lysis buffer (Beyotime) containing protease inhibitors cocktail (ThermoFisher Scientific), and the lysate was centrifugation at 13 700 *g* for 15 minutes. Equal amount of protein was loaded and separated on 10% sodium dodecyl sulfate poly-acrylamide gels and transferred to nitrocellulose membranes. Western blotting analysis was performed with the primary antibodies, including anti-OX2R (1:2000; Abcam), anti-TH (1:2000; Millipore), anti-GAPDH (1:5000; Affinity, USA), and the secondary peroxidase-affinipure goat anti-rabbit IgG (1:10 000Proteintech). We used GAPDH for the normalization of target proteins.

### Perivascular adipose tissue of the aorta and pericardial adipose tissue sample harvest

For pericardial adipose tissue (PAT), after euthanization, the chest was opened, and pericardial adipose tissue fat was peeled off from the heart for later analysis. For perivascular  adipose  tissue  of  the aorta  (PVAD), the aortas of mice were removed, and the perivascular adipose tissue was dissected along the whole aorta and snap-frozen by liquid nitrogen.

### Real-time RT-PCR

Total RNA was isolated from cells or tissues by Trizol reagent (Vazyme, China). Total mRNAs were reverse-transcribed into cDNA for subsequent analyses using the HifairⅡ1st Strand cDNA Synthesis Kit (Yeasen). Real-time RT-PCR (qRT-PCR) was conducted using SYBR Green Master Mix (Yeasen). The expression of target genes was normalized to ribosomal protein S18 (*Rps18*) or glyceraldehyde-3-phosphate dehydrogenase (*Gapdh*) or Hypoxanthine guanine phosphoribosyl transferase (*Hprt*) and quantified with the 2^−ΔΔCT^ method. Sequences of primers for qRT-PCR were listed in ***Table 1***.

**Table 1:** Primers used for real-time RT-PCR

Genes	Sense primer (5′-3′)	Antisense primer (5′-3′)
*Ucp1*	GGCCTTTTTCAAAGGGTTTGT	CAACGGAGCTGTTCATTTGATTT
*Pparγ*	TGTGAGACCAACAGCCTGAC	TCACCGCTTCTTTCAAATCTTGT
*Cidea*	AGGCCGTGTTAAGGAATCTGC	AGCCTATAACAGAGAGCAGGGT
*Adrb3*	AGTCCACCGCTCAACAGGTTT	CAACCAGTCAAGAAGATGGGGA
*Atgl*	TTCGCAATCTCTACCGCCTC	AGCAAAGGGTTGGGTTGGTT
*Hsl*	GGCAAGATCAAAGCCTCAGC	ACATTAGACAGCCGCCGTG
*Lpl*	ACATTCCCTTCACCCTGCCC	GCTGAAGTAGGAGTCGCTTATCC
*Cfd*	CCCGAGGCCGGATTCT	GTCGTCATCCGTCACTCCA
*Adipoq*	CTGACGACACCAAAAGGGCT	AACGTCATCTTCGGCATGACT
*Hcrtr2*	TGGTGCCGACAGATTCCC	TTTCCTTCGTGCTCGGATCT
*Rps18*	CTCTTCCACAGGAGGCCTACACG	TGGCCAGAACCTGGCTATACTTCC
*Gapdh*	CTCCCACTCTTCCACCTTCG	CCTCTCTTGCTCAGTGTCCT
*Hprt*	CAGTCCCAGCGTCGTGATTA	AGCAAGTCTTTCAGTCCTGTC

### Statistical analysis

Statistical analyses were performed using two-tailed, unpaired t-tests or two-way analysis of variance (ANOVA) as indicated in figure legend with GraphPad Prism 9 (GraphPad Software, USA). All data are presented as means with standard error of the mean (SEM). *P*-values less than 0.05 were considered statistically significant.

## Results

### OX2R agonist OB-Ala reduced mice energy expenditure

**Figure 1:**
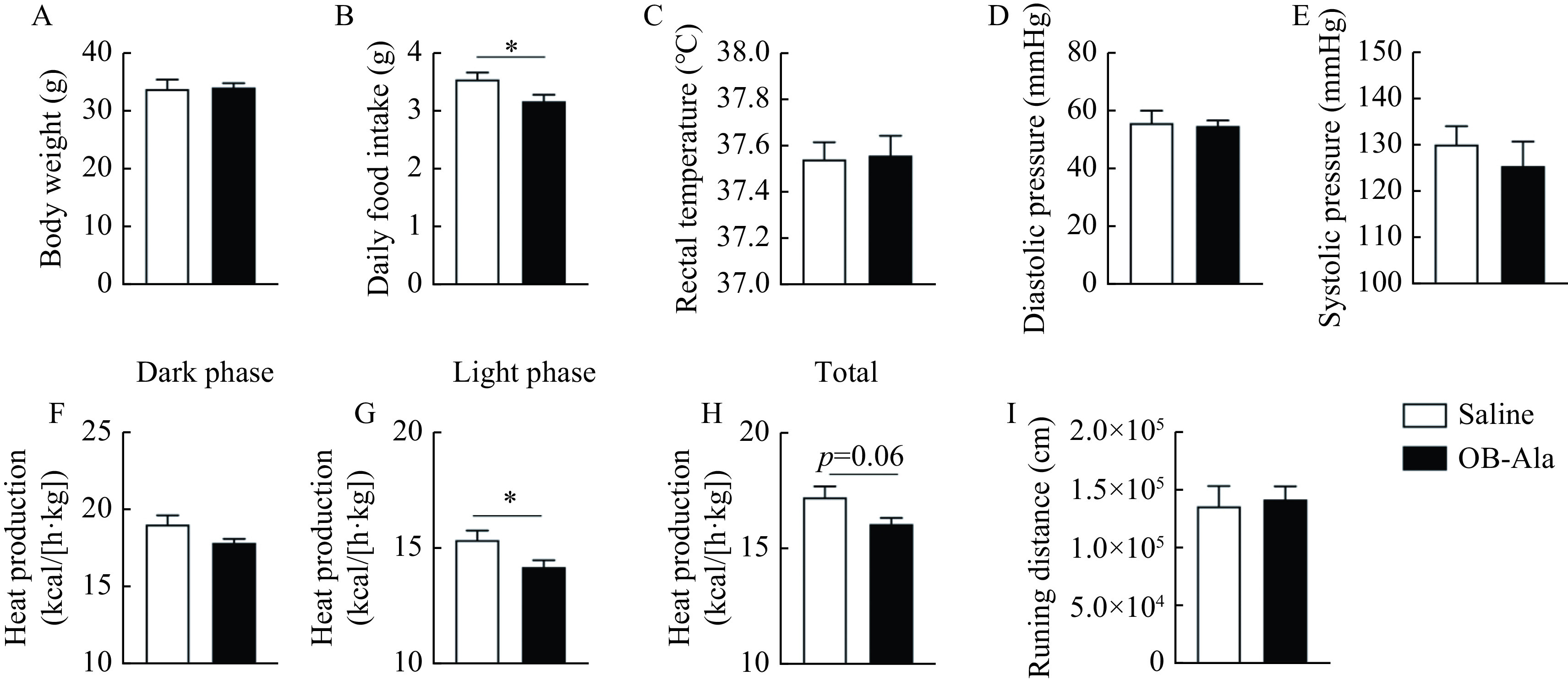
OX2R agonist OB-Ala reduced energy expenditure and food intake.

To explore the effect of the OX2R agonist on systemic metabolism, saline and an OX2R specific agonist OB-Ala were given to C57/BL6J animals by once-daily i.p. injection for 3 weeks. There was no difference in bodyweight between the two groups (***Fig. 1A***) although, averaged daily food intake was lower in the OB-Ala group (***Fig. 1B***). The rectal core temperature did not differ between the two groups at room temperature (***Fig. 1C***).

Diastolic blood pressure and systolic blood pressure were also measured at the end of the study without a difference between the two groups (***Fig. 1D*** and ***E***). Energy expenditure represented by heat production was similar between the two groups in the dark phase (***Fig. 1F***) but significantly lower for OB-Ala treated mice during the light phase (***Fig. 1G***), with a clear trend to decrease in OB-Ala treated mice in total (***Fig. 1H***). The locomotor activity was similar between groups (***Fig. 1I***). Therefore, continuous administration of OX2R agonists reduced the energy expenditure during the light phase in C57/BLJ animals.

### OB-Ala inhibited thermogenesis of brown adipose tissue without affecting white adipose tissue

**Figure 2:**
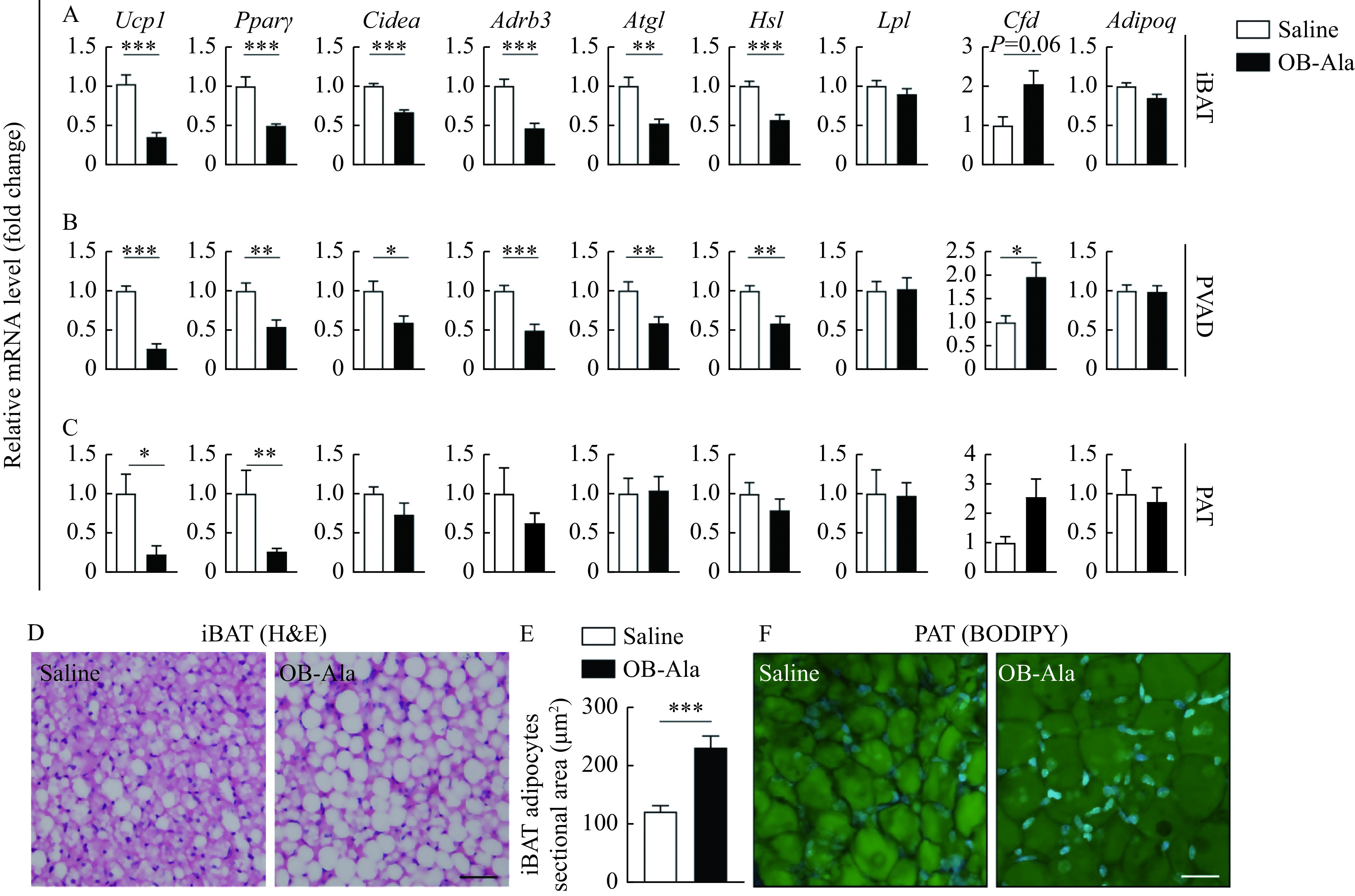
OB-Ala inhibited thermogenesis of brown adipose tissue.

Thermogenesis is an essential component of total energy expenditure, and BAT is the major thermogenic organ in mammals. Therefore, we next assessed BAT functions in OB-Ala treated mice. We first checked the classical brown fat—iBAT, representing the most important brown fat depot. The mRNA levels of the genes encoding BAT specific thermogenic markers like uncoupling protein 1 (*Ucp1*), peroxisome proliferators-activated receptor γ (*Pparγ*), and cell death-inducing DNA fragmentation factor alpha-like effector A (*Cidea*) were all significantly decreased in the OB-Ala group (***Fig. 2A***). β-3 adrenoceptor (*Adrb3*) that mediates sympathetic outflow to BAT was markedly reduced in the OB-Ala group (***Fig. 2A***).

The downregulated expressions of the genes encoding adipose triglyceride lipase (*Atgl*) and hormone-sensitive lipase (*Hsl*) in the OB-Ala group suggests less lipid mobilization (***Fig. 2A***). Expression of the genes for lipoprotein lipase (*Lpl*) and adiponectin (*Adipoq*) did not differ between the two groups (***Fig. 2A***). *Cfd* had a trend to be increased in the OB-Ala group (***Fig. 2A***). All of these data indicate a BAT "whitening" phenomenon after OB-Ala treatment, which is consistent with reduced energy expenditure observed before. Since iBAT is not the only brown fat depot, we also examined PVAD and PAT which are composed of both brown and white fat^[19]^. We found that the thermogenic markers, lipid metabolism enzymes, and adipokines expression patterns in PVAD are almost the same as iBAT (***Fig. 2B***). In PAT, only *Ucp1* and *Pparγ* were downregulated in OB-Ala treated group (***Fig. 2C***).

Histochemical staining results show that the sectional area of adipocytes in iBAT became larger in OB-Ala treated mice (***Fig. 2D*** and ***E***). In PAT, adipocyte morphology was observed in whole-mount samples, and cell size is also larger in OB-Ala groups (***Fig. 2F***), just as the iBAT. Gene expression data and histological data all support that OB-Ala inhibits the thermogenesis of brown adipose tissue and induces a whitening phenotype.

**Figure 3:**
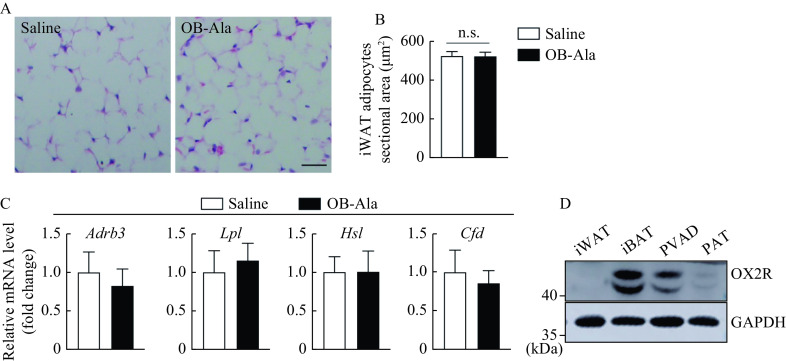
OX2R is selectively expressed in brown adipose tissue and does not affect white adipose tissue.

Next, we investigated whether OB-Ala exerted the same effects in iWAT. Interestingly, the sectional area of adipocytes did not look different between the two groups (***Fig. 3A*** and ***B***). Ucp1 was undetectable in both groups (data not shown). *Adrb3*, *Lpl*, *Hsl*, and *Cfd* were not changed by OB-Ala either (***Fig. 3C***). We wonder whether this discrepancy was due to the OX2R expression pattern in different tissue, therefore, we checked the OX2R protein levels by Western blotting in these fat depots. Indeed, the protein expression level of OX2R was highest in iBAT, followed by PVAD and PAT, and almost undetectable in iWAT (***Fig. 3D***).

### OB-Ala repressed sympathetic innervation of iBAT and POMC neurons in the hypothalamus

**Figure 4:**
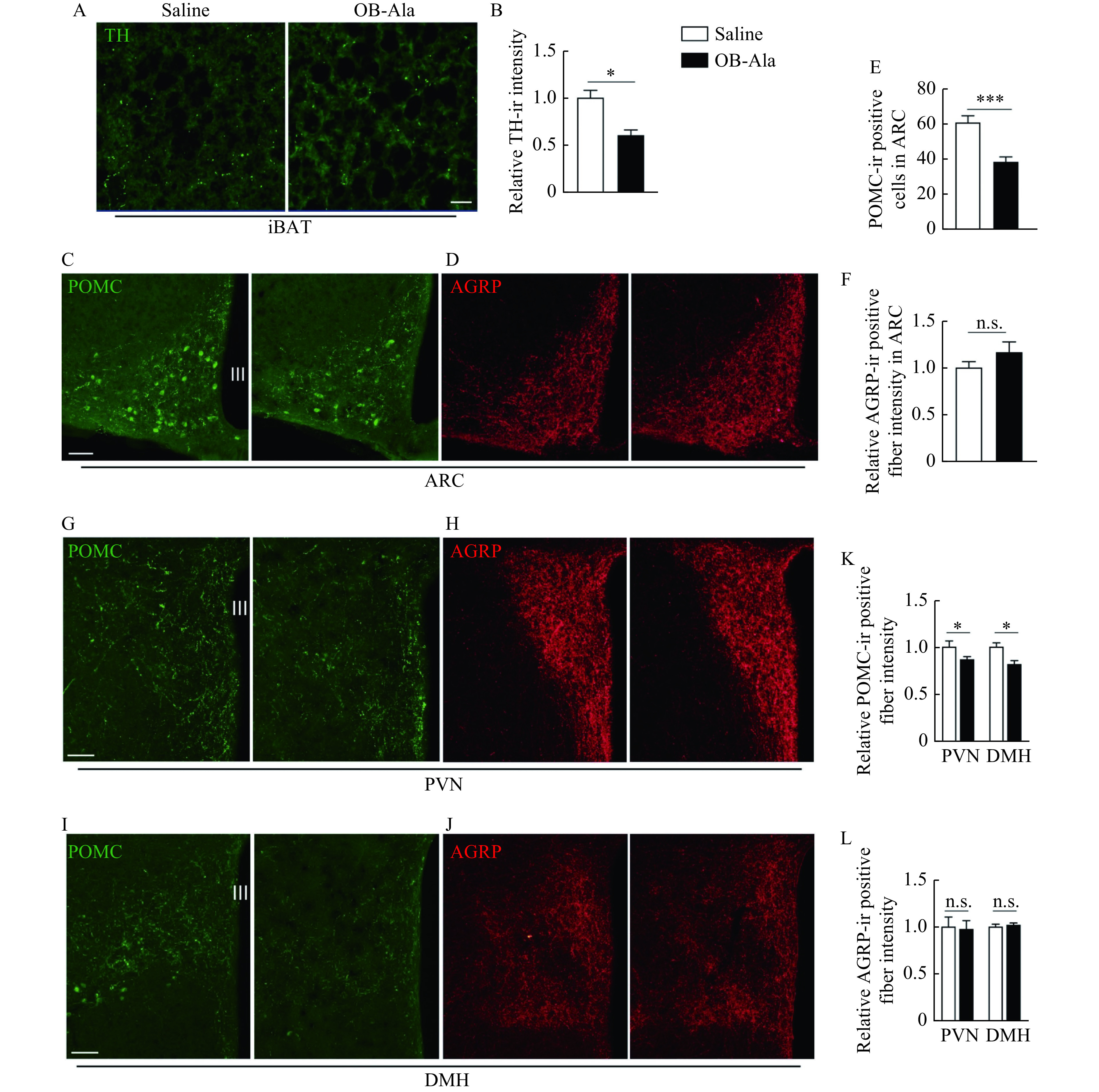
OB-Ala reduced sympathetic innervation of iBAT and POMC expression in the hypothalamus.

To test whether OB-Ala's inhibitory effect on thermogenesis is due to less sympathetic innervation, the density of the sympathetic fibers labelled by TH antibody was examined in iBAT. As expected, TH immunoreactive positive sympathetic fibers density was significantly reduced in iBAT from OB-Ala treated mice (***Fig. 4A*** and ***B***).

The hypothalamus is the region of the brain which controls metabolism and thermogenesis, AGRP and POMC neurons located in the ARC and their projections to the DMH and PVN of the hypothalamus^[20]^. We therefore investigated whether there were consistent changes in the hypothalamus. In the ARC, POMC neurons number was less in brain sections from OB-Ala treated mice (***Fig. 4C*** and ***E***), while the AGRP levels were not different between the two groups (***Fig. 4D*** and ***F***).

In the PVN and DMH, POMC immunoreactivity positive fibers were also less in the OB-Ala group (***Fig. 4G***, ***I*** and ***K***). AGRP fibers density in PVN and DMH did not differ between groups ***(Fig. 4H***, ***J*** and ***L***). Meanwhile, the chronic infusion of OB-Ala did not affect the OX2R expression level in iBAT or hypothalamus (***">Supplementary Fig. 1***, available online). Together, these data suggest that the OX2R agonist OB-Ala reduces the iBAT thermogenesis *via* suppressing the sympathetic innervation of iBAT, and such effect is mediated by a top-down neural pathway from the hypothalamus.

### OB-Ala effect was mediated by afferent neurons instead of brown adipocytes

**Figure 5:**
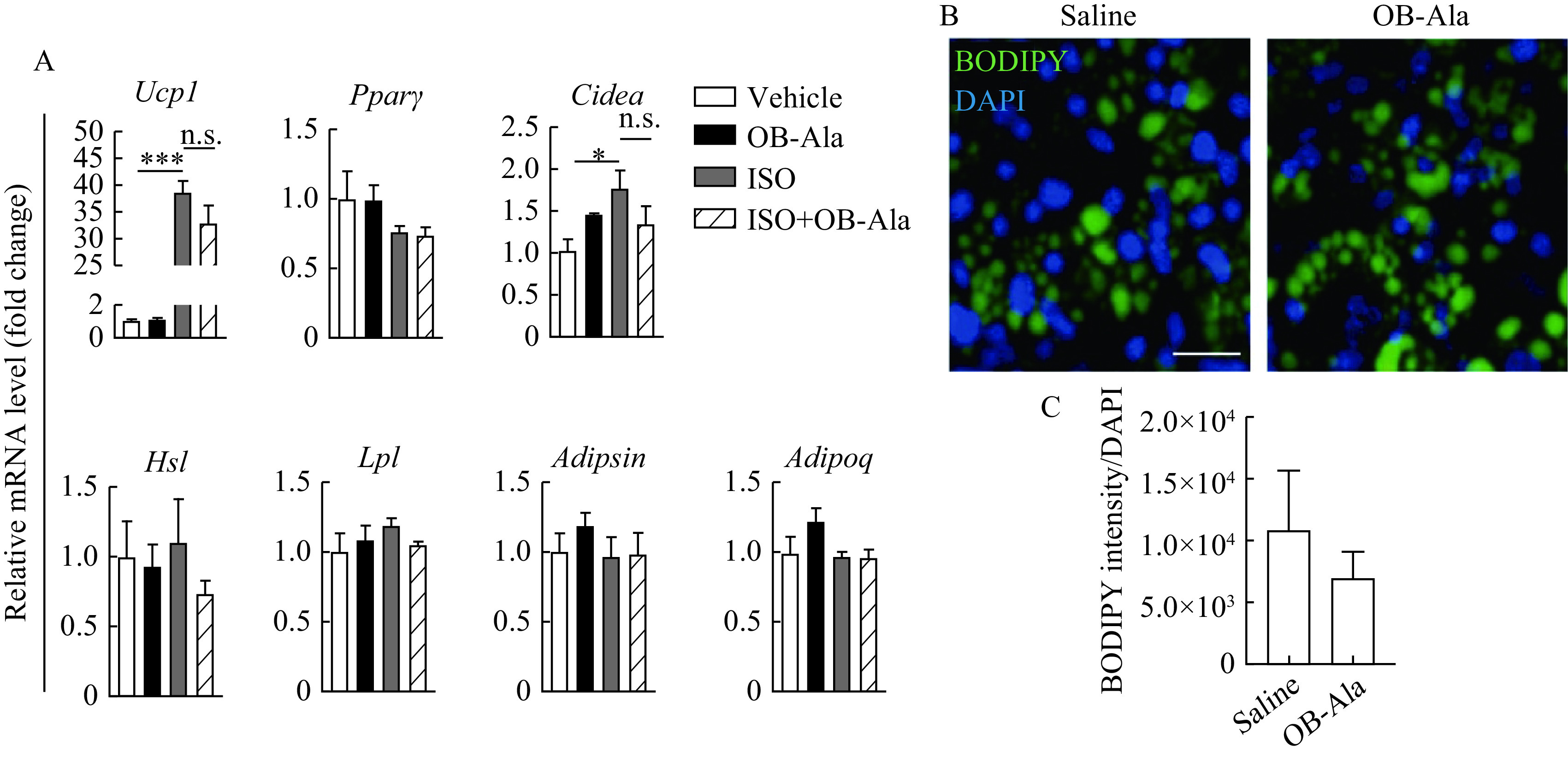
OB-Ala had no effect on thermogenic genes in primary brown adipocytes.

We next investigated whether OB-Ala could directly affect brown adipocytes. We isolated brown adipocyte precursors and differentiated them into mature brown adipocytes. ISO was employed to mimic sympathetic stimulation. Surprisingly, ISO-induced thermogenic responses in brown adipocytes were not attenuated by OB-Ala as indicated by thermogenic markers *Ucp1* and *Cidea*(***Fig. 5A***). Lipid metabolism was not influenced, perhaps due to the short incubation time (4 hours).

OB-Ala did not influence thermogenic genes or lipid metabolism-related genes in the absence of ISO. Lipid droplets contained in brown adipocytes were not changed by OB-Ala either (***Fig. 5B*** and ***C***). These data indicate that OB-Ala may not exert its action directly in brown adipocytes.

**Figure 6:**
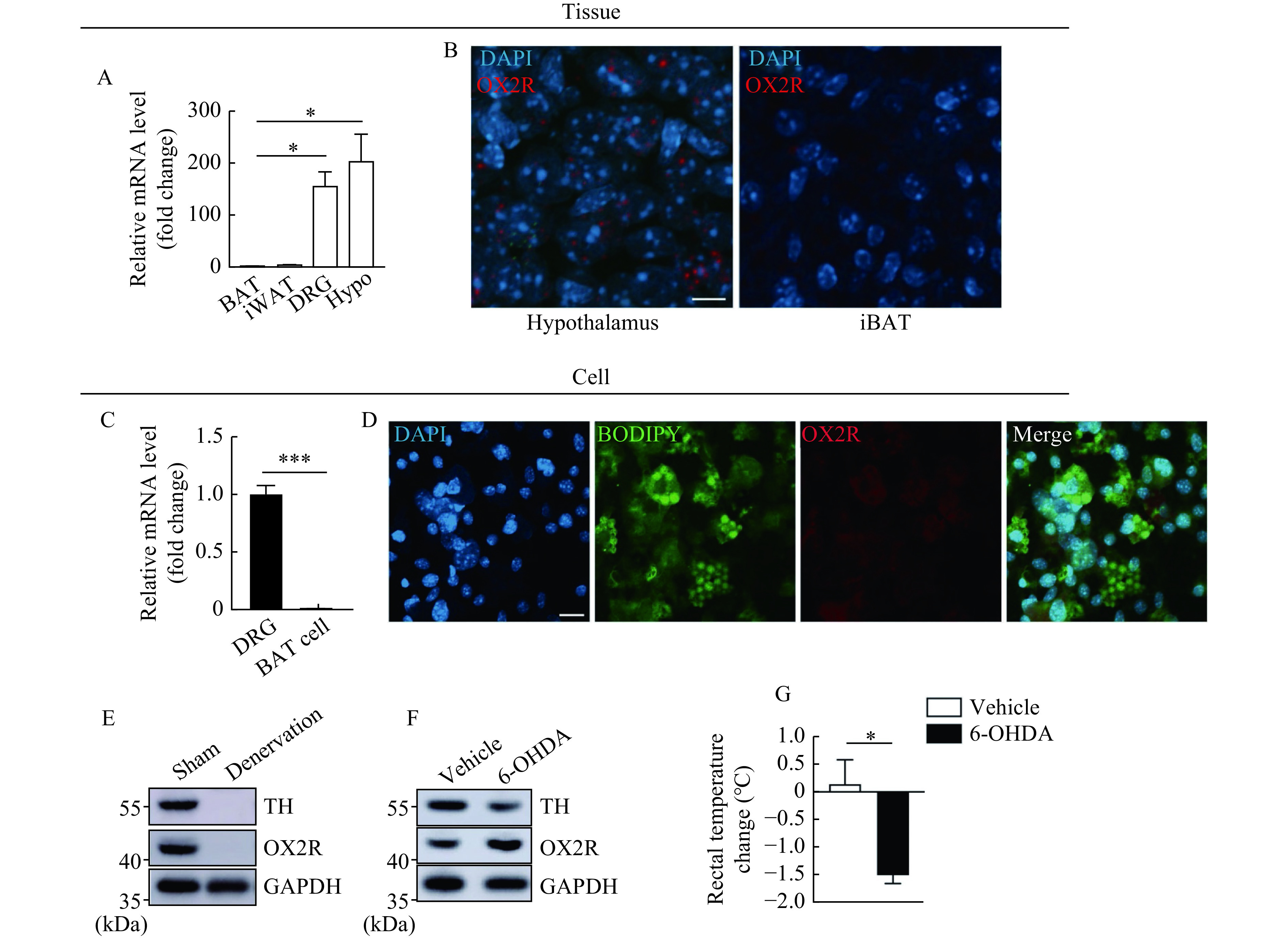
OX2R is located at afferent nerve endings innervating brown adipose tissue.

To identify the exact primary action site of OB-Ala, we examined the expression of OX2R in all the possible tissue and cells at both mRNA and protein levels like iBAT, iWAT and DRG. Hypothalamus tissue was included as the positive reference. Interestingly, we found that mRNA expression of OX2R (*Hcrtr2*) is very low in BAT and iWAT, but relatively high in DRG samples (***Fig. 6A***). *In situ* hybridization by RNAscope had no signal in iBAT either (***Fig. 6B***). In cultured brown adipocytes and primary DRG neurons, we also found that mRNA of OX2R was barely detected in brown adipocytes but quite high in neurons isolated from DRG (***Fig. 6C***). *In situ* hybridization did not yield any signals in primary brown adipocytes (***Fig. 6D***).

To confirm that OX2R was initially expressed in neurons in DRG, we did iBAT denervation surgery to confirm this hypothesis by cutting off all the nerves innervating iBAT and compared the OX2R protein expression by Western blotting. As shown in ***Fig. 6E***, the disappearance of TH suggests sufficient denervation. OX2R was also disappeared after denervation surgery. This result indicates a neuronal origin of OX2R protein in iBAT, though the afferent and efferent nerves could not be differentiated. To clarify this issue, 6-OHDA was used to destroy iBAT sympathetic nerves, specifically. Two weeks after injection, TH in iBAT was decreased in 6-OHDA injected mice (***Fig. 6F***).

The TH levels in the adrenal, iWAT and ventral tegmental area (VAT) were examined to ensure the 6-OHDA was not leaked out from the iBAT (***Supplementary Fig. 2***, available online). The 6-OHDA injected mice could not maintain their core temperature after cold exposure (***Fig. 6G***), suggesting the success of sympathetic denervation. In this scenario, the OX2R protein level was not decreased in the denervated group (***Fig. 6F***). This data further supports our speculation that the OX2R proteins detected in iBAT are originated from afferent nerves instead of brown adipocytes.

### Acute OB-Ala infusion inhibited iBAT thermogenesis during cold exposure

**Figure 7:**
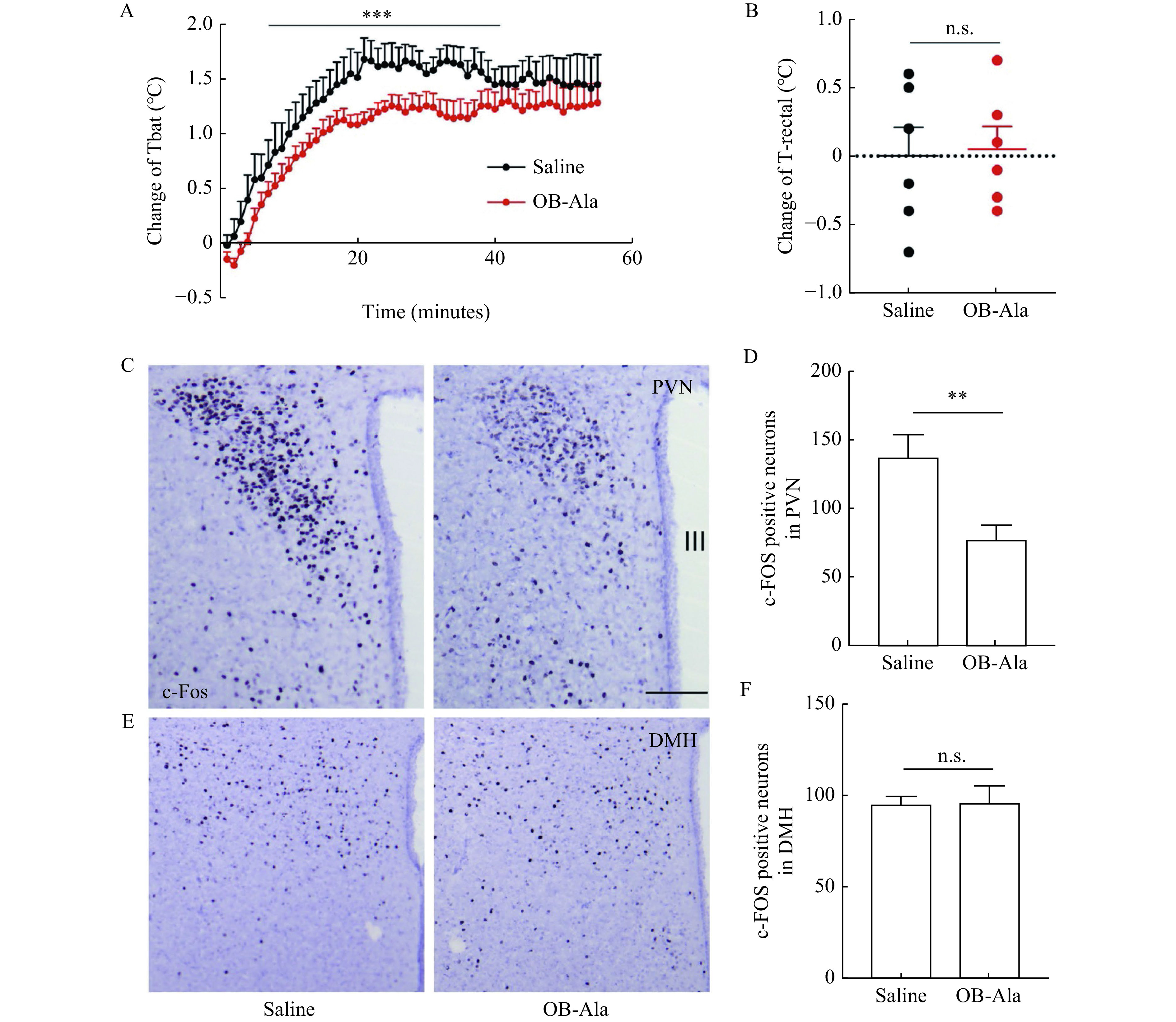
Intra-BAT infusion of OB-Ala inhibited iBAT thermogenesis during cold exposure.

To further test whether iBAT afferent nerves are the primary action sites of OB-Ala, we did microinjection of OB-Ala directly into iBAT and assessed the iBAT temperature of these animals subjected to 60 minutes cold exposure in a 4-degree chamber. The temperature change of iBAT quickly increased for the first 20 minutes and reached the plateau for the rest of the time. The change of iBAT temperature was significantly lower in OB-Ala injected animals (***Fig. 7A***). The core (rectal) temperature did not differ between the two groups (***Fig. 7B***).

We also compared the neuronal activity in PVN and DMH, the key regions controlling sympathetic output and BAT thermogenesis. As indicated by c-Fos staining, the neuronal activity was markedly elevated by cold exposure and attenuated by OB-Ala microinjection in PVN (***Fig. 7C*** and ***D***). The c-Fos positive cell numbers were not different between the two groups in DMH (***Fig. 7E*** and ***F***). To exclude the possibility that peripheral OB-Ala penetrated into the brain and caused a direct inhibitory effect on PVN neurons, we did i.c.v. injection of OB-Ala and found that OB-Ala could not directly decrease c-Fos levels in PVN and DMH, even with a trend to increase c-Fos levels in PVN (***Supplementary Fig. 3***, available online). These data suggest that the thermogenic inhibitory effect of OB-Ala is mediated through afferent nerves of iBAT to PVN, which finally decreases the sympathetic feedback activities.

## Discussion

Orexin is an appetite-promoting neuropeptide. The functions of orexin signaling mediated *via* its two receptors are far more than feeding. OX2R is implicated as an important therapeutic target for narcolepsy and obesity^[21]^. Most data are based on loss of function animal models or central infusion of pharmacological agonists or antagonists. Our study observed decreased energy expenditure in animals subjected to chronic i.p. injection of an OX2R specific agonist, which ends up with an opposite phenotype compared to central infusion of OX2R agonist. This suppressed energy expenditure was further supported by the decreased thermogenic markers and enlarged brown adipocytes size in BAT. These surprising findings suggest that i.p. injected OX2R agonist may exert its action on energy expenditure *via* a different mechanism besides central nervous system (CNS) signaling. In chronic injection experiments, the bodyweight of animals does not change with OB-Ala treatment. This is perhaps due to the decrease of both energy expenditure and food intake.

A series of studies reported that infusion of OXR agonists in the brain (either by i.c.v. or in specific brain regions) could evoke sympathoexcitatory responses and increase cardiovascular activities^[22–24]^. Enhanced energy expenditure was also observed in mice that received central OX2R agonist infusion^[7]^. In contrast, our study found that energy expenditure and sympathetic innervation of BAT was decreased in OB-Ala injected animals, indicating that peripheral OX2R signal may exert a counter-regulation effect on energy expenditure. Although, orexin was named according to its orexigenic effect when discovered, later studies proved that chronic i.c.v. infusion of orexin-A does not change food intake, and two weeks i.c.v. infusion of OB-Ala could even suppress food consumption in high fat diet-fed animals^[7,25]^. Since both central and peripheral mechanisms may contribute to the phenotypes of i.p. administration of OB-Ala, we could not explicitly explain the mechanism behind the decreased food intake.

Whether OX2R is expressed in adipocytes and whether orexin directly affects adipocytes is a controversial issue^[2,8,26–27]^. To clarify, we examined the protein and mRNA expression of OX2R at both cell and tissue levels. We first found that OX2R is selectively expressed in BAT but not in iWAT. Although, a few reports claim that orexin could stimulate the differentiation of brown adipocytes, we could barely detect the mRNA expression of OX2R in cultured brown adipocytes. Also, as expected, the OB-Ala treatment does not suppress the thermogenic responses induced by ISO. In further iBAT denervation experiments, we confirmed that the OX2R proteins detected in iBAT are not derived from adipocytes but the nerve endings.

It is well known that thermogenesis by brown adipose tissue is regulated by sympathetic nerves^[28–29]^. Norepinephrine released from the sympathetic nerve endings triggered thermogenesis *via* the Adrb3. We observed the downregulation of Adrb3 and less sympathetic nerve endings in iBAT of OB-Ala injected mice, making us doubt whether OX2R is located at sympathetic nerve endings. We employed a sympathetic nerve denervation experiment to confirm that OX2R is not located on efferent nerves but on afferent nerves. Brown adipose tissue also has sensory innervation, contributing to thermogenesis regulation. Our data also confirmed that the OX2R mRNA level is abundant in DRG neurons, almost comparable to the hypothalamus. Therefore, we speculate that DRG is the primary action site of i.p. injected OB-Ala.

It is well-documented that preoptic area (POA) and DMH are also key nuclei controlling efferent output to BAT in the thermoregulation^[30]^. However, in our cold exposure study, we found a significant less c-Fos signal in PVN but not in DMH in OB-Ala treated group. We speculate that such discrepancy may be due to different afferent pathways. Thermoregulation relies on feedforward sensory signals from both cutaneous nerves and visceral nerves. A large amount of literature reported that POA integrates signaling from cutaneous thermoreceptors and projects to DMH to initiate efferent pathways^[30]^. However, less is known about the role of thermosensory information from visceral afferent input in the thermoregulation.

Ryu *et al* depicted afferent and effect pathways of iBAT in a virus tracing study with both retrograde and anterograde transneuronal viral tract tracers. They confirmed that PVN is the primary integration center in iBAT sympathetic-sensory feedback circuit^[31]^. In our study, OB-Ala was directly injected into iBAT, which may only inhibit the conduction of visceral afferent of iBAT without affecting cutaneous afferent input to POA and DMH. Meanwhile, the reduced sympathetic outflow was only observed in brown fat but not in white fat. We speculate that a precise feedback regulation mechanism might be based on the sensory input location. Evidence from literature also supports that different fat pads own unique sympathetic nodes^[32]^. The detailed mechanism needs further investigation.

In summary, this study revealed a novel mechanism by which OX2R signaling regulates iBAT thermogenesis. OX2R is expressed in afferent nerve endings innervating iBAT instead of brown adipocytes. OX2R agonist could inhibit sensory input from iBAT during cold exposure and therefore dampens the sympathetic-sensory circuit of iBAT to reduce thermogenesis. These results also suggest that OX2R signaling contains a feedback loop between iBAT and brain, which may provide clues for the explanation of complex phenotypes in the orexin study field.
